# High-speed C–H chlorination of ethylene carbonate using a new photoflow setup

**DOI:** 10.3762/bjoc.18.16

**Published:** 2022-01-27

**Authors:** Takayoshi Kasakado, Takahide Fukuyama, Tomohiro Nakagawa, Shinji Taguchi, Ilhyong Ryu

**Affiliations:** 1 Organization for Research Promotion, Osaka Prefecture University, Sakai, Osaka 599-8531, Japan; 2 Department of Chemistry, Graduate School of Science, Osaka Prefecture University, Sakai, Osaka 599-8531, Japan; 3 Wakayama Research & Development Group, Nankai Chemical Co. Ltd., 1-1-38 Kozaika, Wakayama 641-0007, Japan; 4 Department of Applied Chemistry, National Yang Ming Chiao Tung University (NYCU), Hsinchu 30010, Taiwan

**Keywords:** C–H chlorination, chlorine gas, ethylene carbonate, photo flow reactor, vinylene carbonate

## Abstract

We report the high-speed C–H chlorination of ethylene carbonate, which gives chloroethylene carbonate, a precursor to vinylene carbonate. A novel photoflow setup designed for a gas–liquid biphasic reaction turned out to be useful for the direct use of chlorine gas. The setup employed sloped channels so as to make the liquid phase thinner, ensuring a high surface-to-volume ratio. When ethylene carbonate was introduced to the reactor, the residence time was measured to be 15 or 30 s, depending on the slope of the reactor set at 15 or 5°, respectively. Such short time of exposition sufficed the photo C–H chlorination. The partial irradiation of the flow channels also sufficed for the C–H chlorination, which is consistent with the requirement of photoirradiation for the purpose of radical initiation. Near-complete selectivity for single chlorination required the low conversion of ethylene carbonate such as 9%, which was controlled by limited introduction of chlorine gas. At a higher conversion of ethylene carbonate such as 61%, the selectivity for monochlorinated ethylene carbonate over dichlorinated ethylene carbonate was 86%. We found that the substrate contamination with water negatively influenced the performance of the C–H chlorination.

## Introduction

**Scheme 1:**
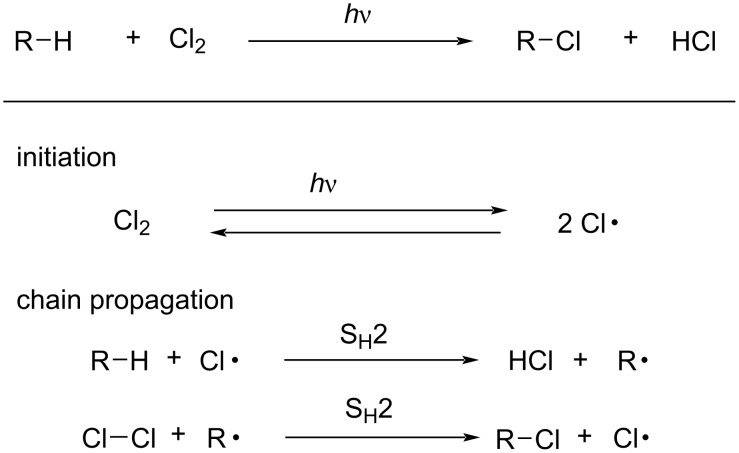
Radical chain mechanism for a photo-induced C–H chlorination reaction.

The C–H chlorination by molecular chlorine is a highly exothermic reaction that proceeds via a radical chain mechanism as illustrated in Scheme 1 [1–6]. Frequently, photoirradiation is used for radical initiation through homolysis of the Cl–Cl bond to generate chlorine radicals. In a subsequent step, a S_H_2 reaction by chlorine radicals at C–H bonds generates alkyl radicals and HCl. The second S_H_2 reaction between alkyl radicals and molecular chlorine then occurs to give the C–H chlorinated product and a chlorine radical, sustaining the radical chain. Chlorine gas is a cheap feedstock since it is formed as a byproduct of the electrolysis of NaCl to produce NaOH in an industrial process [7]. We felt that C–H chlorination would be updated by using scalable flash chemistry [8].

**Figure 1:**
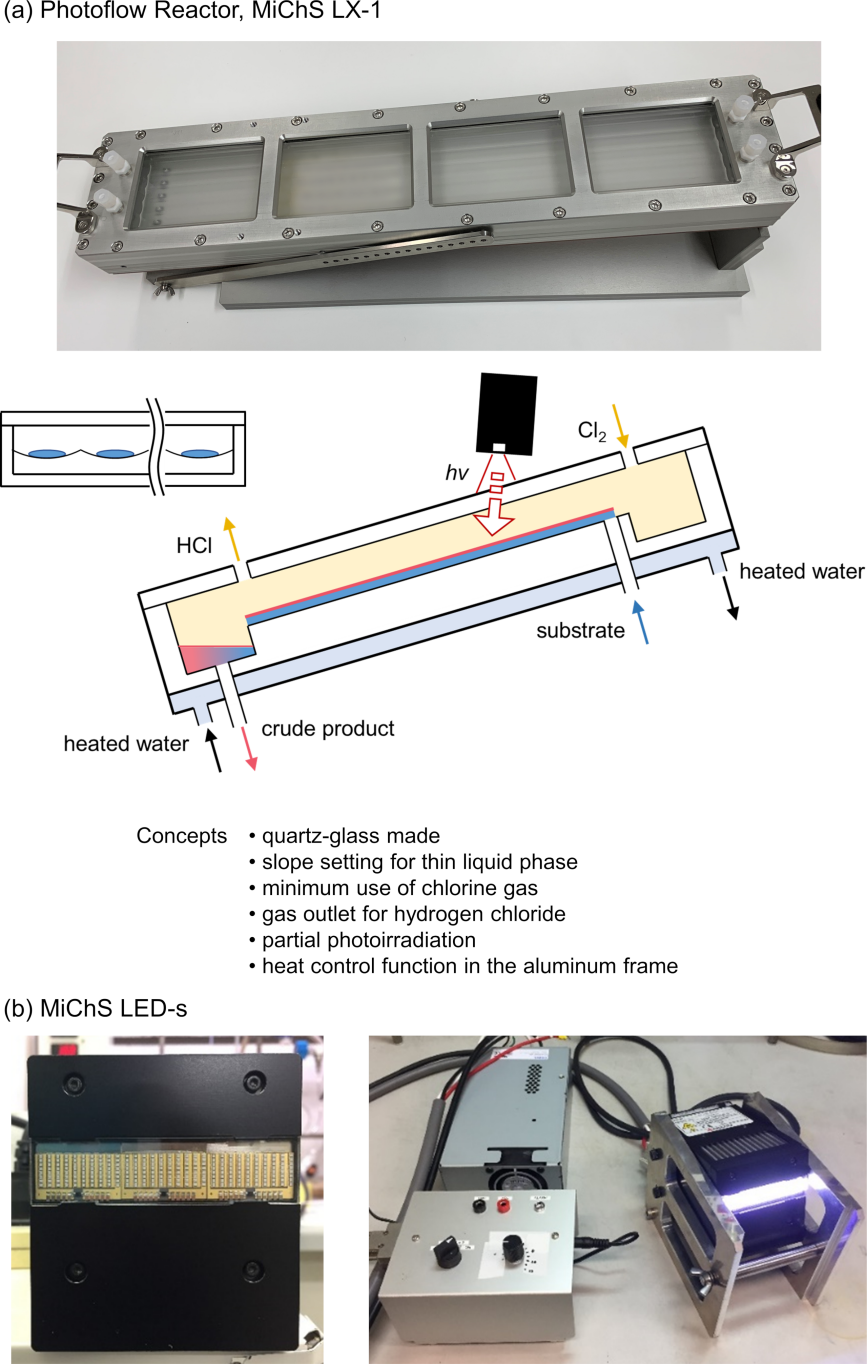
Components for photoflow setup: (a) MiChS LX-1 reactor and (b) MiChS LED-s (365 ± 5 nm, 60–600 W).

Flow C–H chlorination using a compact flow reactor is highly desirable in terms of efficiency and safety in handling highly toxic gases such as chlorine. In 2002, Jähnisch and co-workers reported the first microflow chlorination of 2,4-diisocyano-1-methylbenzene, which used a falling-film reactor developed by IMM [9]. While the flow rate employed was quite low (0.12 mL/min of toluene), the residence time was less than 14 seconds. More recent studies on flow C–H chlorination reactions focused on the use of Cl_2_ gas in situ generated by photolysis of sulfuryl chloride [10] or by acid treatment of NaOCl [11–12]. We thought that if rationally designed scalable photoflow setups were available, flow C–H chlorination reactions using chlorine gas would be able to focus on production. In this study, we tested a novel photoflow setup consisting of quartz-made straight-line reactors, which are provided from MiChS (LX-1, Figure 1a) and a high-power LED (MiChS LED-s, 365 ± 5 nm, Figure 1b) [13]. Each channel track has a 2 mm depth and 557 mm length, while the width varies from 6 or 13 mm depending on the number of channels 7 or 5, respectively. The flow photoreactor is embedded into an aluminum frame equipped with a heat carrier channel. The design concepts including angle settings to ensure a thin liquid layer are summarized in Figure 1.

**Scheme 2:**
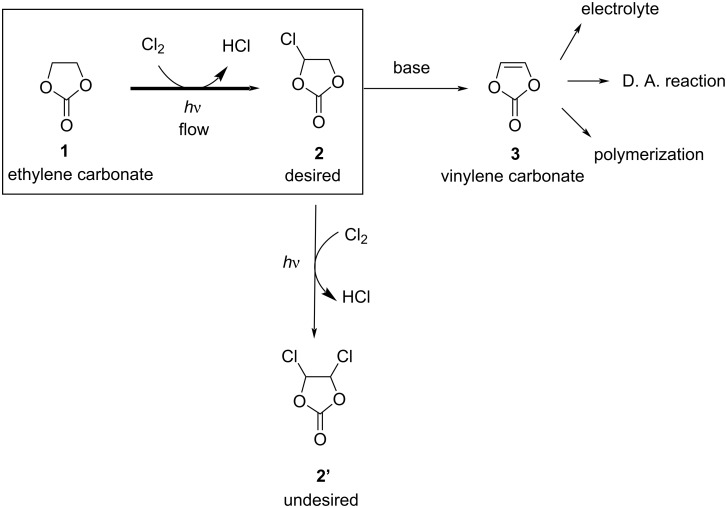
Model reaction: photoflow C–H chlorination of ethylene carbonate (**1**) to chloroethylene carbonate (**2**).

We chose the C–H chlorination of ethylene carbonate (**1**) as a model reaction (Scheme 2). Chlorinated ethylene carbonate **2** is a precursor to vinylene carbonate (**3**), which is used as an electrolyte additive for Li-ion batteries [14–20]. Vinylene carbonate also serves as a useful synthetic building block for Diels–Alder reactions [21–25] and polymerization [26–30].

## Results and Discussion

**Figure 2:**
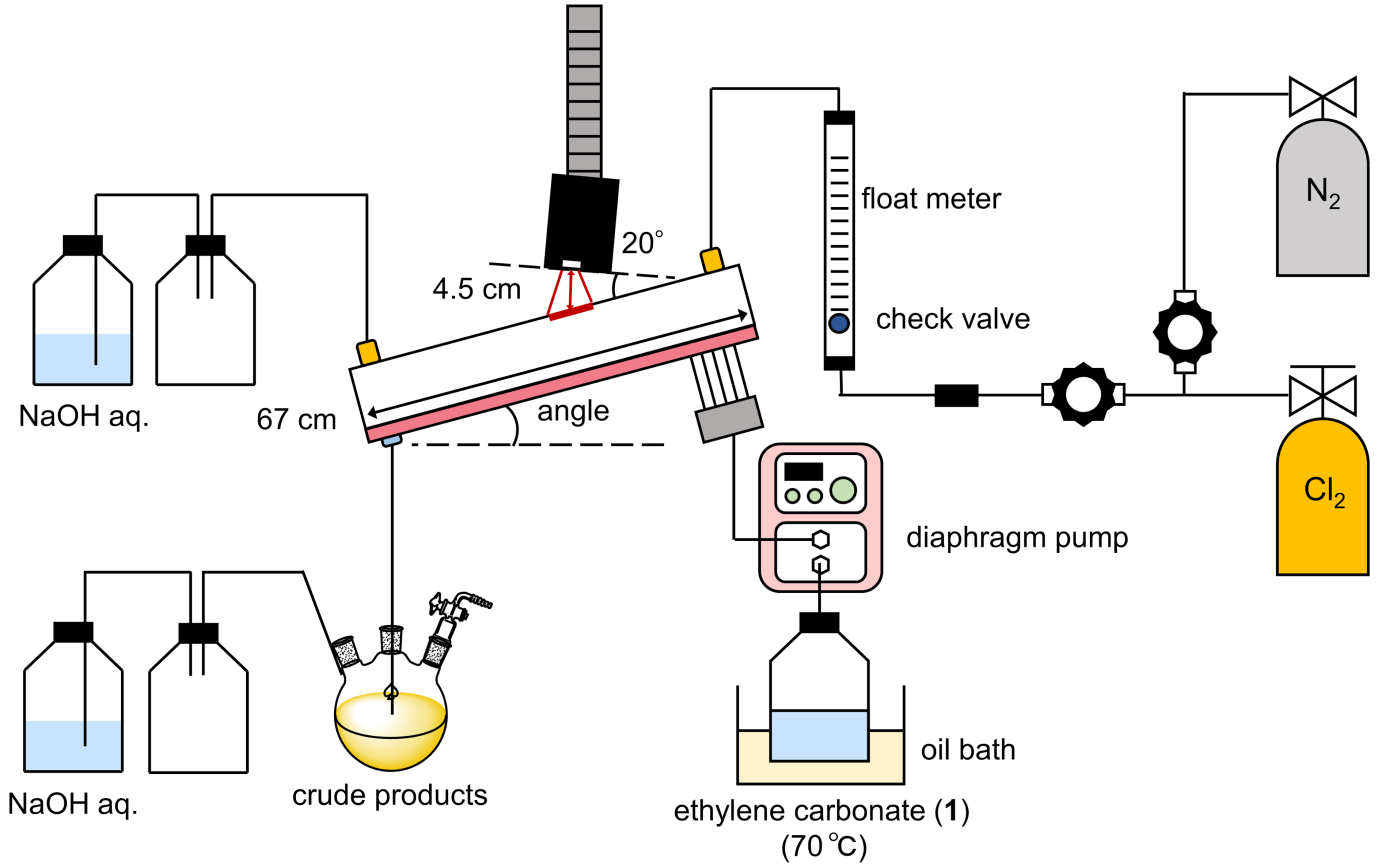
Photoflow setup for the C–H chlorination of ethylene carbonate (**1**).

Using a PTFE tube and PTFE connectors, we connected the photoflow setup with a chlorine gas cylinder through a floating gas level meter in a fume hood (Figure 2). Since ethylene carbonate (**1**) melts between 34–37 °C, we preheated the container of **1** using an oil bath at 70 °C and pumped it to the photoreactor. In the reactor, hot water (80 °C) was circulated through a hole channel manufactured in an aluminum-made frame to keep the contacted glass reactor warm. The LED lamp was placed on the upper side of the reactor with a 20° angle to the reactor surface. The exiting gases (HCl and unreacted Cl_2_) were trapped by an aqueous NaOH solution (1.7 M).

The reactors are set with a slope of 15 or 5° to achieve a thin substrate layer causing a rapid gas/liquid biphasic reaction. The residence time was estimated to be 15 and 30 seconds, respectively (for the measurement, ethylene carbonate was introduced in the absence of chlorine gas). After the experiments, chlorine gas that remained inside the flow setup was flushed with N_2_ gas. In general, we used ethylene carbonate (**1**) with the grade containing less than 0.03% of water. The results are summarized in Table 1.

**Table 1:** Photoflow C–H chlorination of ethylene carbonate (**1**) to chloroethylene carbonate (**2**).^a^

entry	angle (°)	flow rate		UV-LED (W)	conversion (%)^b^	selectivity (%)^b^	
	
		**1**^a^ (mmol/min)	Cl_2_ (mmol/min) (equiv)			**2**	**2’**

1	15	74.9	12.5 (0.17)	240	9	100	0
2	15	74.9	17.4 (0.23)	240	12	96	4
3	15	74.9	33.9 (0.45)	240	21	91	9
4	15	74.9	75.9 (1.01)	240	39	89	11
5^c^	15	74.9	75.9 + 75.9 (2.02)	240	87	74	26
6	15	46.4	91.5 (1.97)	240	61	86	14
7	15	29.6	91.5 (3.09)	240	76	84	16
8	15	117.6	146.5 (1.25)	240	49	78	22
9	15	117.6	143.7 (1.22)	600	47	78	22
10	5	117.6	146.5 (1.25)	240	61	79	21

^a^Reactions were conducted by using LX-1 with a reactor angle of 15° or 5° (entry 10). Photoirradiation was carried out by using LEDs (365 ± 5 nm at the power of 240 or 600 W). Ethylene carbonate (**1**) contains 0.03% of H_2_O. ^b^Determined by GC analysis. ^c^Reaction mixture was circulated twice.

When the reaction of ethylene carbonate (**1**, flow rate: 74.9 mmol/min, containing 0.03% of H_2_O) with 0.17 equiv of Cl_2_ gas (flow rate: 12.5 mmol/min) was carried out under irradiation by UV-LED (240 W) with a 15° reactor angle, the desired chloroethylene carbonate (**2**) was formed selectively with a 9% conversion of **1** (Table 1, entry 1). When 0.23 equiv of Cl_2_ was used, the selectivity became 96% with 12% conversion of **1**, in which a small amount of undesired 1,2-dichloroethylene carbonate (**2’**) was detected by GC (Table 1, entry 2). When 0.45 equiv of Cl_2_ was used, the conversion of **1** increased to 21% and the selectivity of **2** became 91% (Table 1, entry 3). The reaction of **1** with one equivalent of Cl_2_ gave **2** and **2’** in a ratio of 89:11 with 39% conversion of **1** (Table 1, entry 4). When the reaction mixture was circulated twice, we observed a higher conversion of **1** (87%) and obtained a 74:26 mixture of **2** and **2’** (Table 1, entry 5). Then, we limited the feeding of **1** (flow rate: 46.4 mmol/min) in order to increase conversion, which worked well. The reaction of **1** with 1.97 equiv of Cl_2_ resulted in 61% conversion of **1** and an 86:14 ratio of **2** and **2’** (Table 1, entry 6). When a lower feeding of **1** (29.6 mmol/min) and an excess amount of Cl_2_ (3.09 equiv) were used, higher conversion of **1** (76%) was attained with the selectivity of 84:16 (Table 1, entry 7). The irradiation at 600 W gave an almost similar result (Table 1, entries 8 and 9), which suggested that 240 W sufficed the reaction. Indeed, when the reaction was carried out with a shallow reactor angle such as 5°, the conversion of **1** increased from 49 to 61% (Table 1, entries 8 and 10). This is due to the extended residence time from 15 to 30 s. Flow gas/liquid reactions are often carried out using a tubular reactor and mixer under slug flow conditions. However, it is not easy to apply such conditions to the present photochlorination reaction since the volume of the Cl_2_ gas is ca. 400 times larger than that of ethylene carbonate (for entry 8 in Table 1). In addition, a much longer tubular reactor would be required to ensure 15–30 s residence time.

We then investigated the effect of contamination with water on the reaction, since Cl_2_ gas is known to react with H_2_O under irradiation conditions [31] and the results are summarized in Table 2. The flow rate of **1** and the equivalents of chlorine to **1** were set to be 187 mmol/min and 0.60–0.69, respectively. The reactor angle and light power were 15° and 240 W, respectively. The chlorination reaction using an ordinary grade of the substrate **1** containing 0.03% of water gave a 96:4 ratio of products **2** and **2’** with 26% conversion of **1** (Table 2, entry 1). In contrast, when we used substrate **1** containing 0.15% of water, the conversion decreased to 11% (Table 2, entry 2). With 0.76% of water, the conversion decreased further to 9% (Table 2, entry 3). These results suggest that the reaction has to be carried out carefully under dry conditions.

**Table 2:** Effect of contamination of water.^a^

entry	water contamination	flow rate		conversion (%)^b^	selectivity (%)^b^	
	
		**1**^a^ (mmol/min)	Cl_2_ (mmol/min) (equiv)		**2**	**2’**

1	0.03%	187.0	126.8 (0.68)	26	96	4
2	0.15%	187.0	112.7 (0.60)	11	92	8
3	0.76%	187.0	118.3 (0.63)	9	100	0

^a^Reactions were conducted by using LX-1 with a rector angle of 15° and LEDs (240 W). ^b^Measured by GC.

## Conclusion

In this work, we reported that a novel photoflow setup designed for a gas–liquid biphasic reaction turned out to be useful for the C–H chlorination using chlorine gas in flow. Two decades after the first report on the microflow chlorination of a toluene derivative by Jähnisch and co-workers, we propose a new photoflow setup for C–H chlorination using chlorine gas, applicable to scalable flow C–H chlorination. In our test reaction using C–H chlorination of ethylene carbonate (**1**), chloroethylene carbonate (**2**) was obtained in good to excellent selectivity by tuning the flow rates of **1** and chlorine gas. Partial irradiation of the flow channel is sufficient for the C–H chlorination, consistent with the requirement for light irradiation for the radical initiation step. If we apply the conditions to give 80% selectivity with 60% conversion with 30 s residence time, around 15 kilograms of chloroethylene carbonate (**2**) can be synthesized per day, which suggests the high potential of the present photoflow setup. We also demonstrated that the contamination with water had a negative impact on the reaction and the system should be kept dry for continuous production. We are now investigating some other photo gas–liquid flow reactions, which will be reported in due course.

## Experimental

The photoflow setup consisting of a flow photoreactor LX-1 and UV-LEDs were supplied from MiChS Inc., Ltd. (http://www.michs.jp). The angle of the photoflow reactor was set to be 15 or 5° and heated water at 80 °C was circulated in a channel of an aluminum-made frame to avoid solidification of ethylene carbonate (**1**), whose melting point is 34–37 ºC. The UV-LED (365 ± 5 nm) was set with an angle of 20° to the reactor surface. Ethylene carbonate (**1**) preheated to 70 °C was fed into each channel of the flow photoreactor by using a diaphragm pump. At the same time, chlorine gas was fed into the reactor from the top-side inlet. Evolved HCl gas and unreacted Cl_2_ gas were trapped by an aqueous 1.7 M NaOH solution. The first eluted solution was discarded for 3 min after which the eluted solution was collected for analysis. GC analysis was performed on a Shimadzu GC-2014 equipped with an FID detector using an Agilent J&W DB-1 column (Ø 0.25 mm × 30 m) under the following conditions: initial oven temperature: 40 °C, temperature change rate of 5 °C/min to 250 °C, hold at this temperature for 10 min. Yields were determined by using the percentage peak area method with compensation for the relative sensitivities of each component. Product **2** and byproduct **2’** were confirmed by ^1^H and ^13^C NMR analysis (see Supporting Information File 1).

## Supporting Information

File 1 GC analysis and NMR spectra of the crude reaction mixture for the chlorination of compound **1**.

## References

[1] Ingold K U, Lusztyk J, Raner K D (1990). Acc Chem Res.

[2] Fletcher B, Suleman N K, Tanko J M (1998). J Am Chem Soc.

[3] Sun N, Klabunde K J (1999). J Am Chem Soc.

[4] Pease R N, Walz G F (1931). J Am Chem Soc.

[5] Brown H C, Kharasch M S, Chao T H (1940). J Am Chem Soc.

[6] Kharasch M S, Berkman M G (1941). J Org Chem.

[7] Wang Y, Liu Y, Wiley D, Zhao S, Tang Z (2021). J Mater Chem A.

[8] Yoshida J-i (2008). Flash Chemistry: Fast Organic Synthesis in Microsystems.

[9] Ehrich H, Linke D, Morgenschweis K, Baerns M, Jähnisch K (2002). Chimia.

[10] Matsubara H, Hino Y, Tokizane M, Ryu I (2011). Chem Eng J.

[11] Fukuyama T, Tokizane M, Matsui A, Ryu I (2016). React Chem Eng.

[12] Strauss F J, Cantillo D, Guerra J, Kappe C O (2016). React Chem Eng.

[13] Hyodo M, Iwano H, Kasakado T, Fukuyama T, Ryu I (2021). Micromachines.

[14] Zhang S S (2006). J Power Sources.

[15] Ivanov S, Sauerteig D, Dimitrova A, Krischok S, Bund A (2020). J Power Sources.

[16] Michan A L, Parimalam B S, Leskes M, Kerber R N, Yoon T, Grey C P, Lucht B L (2016). Chem Mater.

[17] Liu Y-H, Takeda S, Kaneko I, Yoshitake H, Yanagida M, Saito Y, Sakai T (2016). RSC Adv.

[18] Wang Y, Nakamura S, Tasaki K, Balbuena P B (2002). J Am Chem Soc.

[19] Burns J C, Petibon R, Nelson K J, Sinha N N, Kassam A, Way B M, Dahn J R (2013). J Electrochem Soc.

[20] Xiong D, Burns J C, Smith A J, Sinha N, Dahn J R (2011). J Electrochem Soc.

[21] Aotake T, Tanimoto H, Hotta H, Kuzuhara D, Okujima T, Uno H, Yamada H (2013). Chem Commun.

[22] Geiseler O, Müller M, Podlech J (2013). Tetrahedron.

[23] Revés M, Lledó A, Ji Y, Blasi E, Riera A, Verdaguer X (2012). Org Lett.

[24] Dong S, Cahill K J, Kang M-I, Colburn N H, Henrich C J, Wilson J A, Beutler J A, Johnson R P, Porco J A (2011). J Org Chem.

[25] Taffin C, Kreutler G, Bourgeois D, Clot E, Périgaud C (2010). New J Chem.

[26] Huang X, Wu J, Wang X, Tian Y, Zhang F, Yang M, Xu B, Wu B, Liu X, Li H (2021). ACS Appl Energy Mater.

[27] Zhang Y, Chen S, Chen Y, Li L (2021). Mater Chem Front.

[28] Li H, Yang J, Xu Z, Lu H, Zhang T, Chen S, Wang J, NuLi Y, Hirano S-i (2020). ACS Appl Energy Mater.

[29] Chai J, Liu Z, Zhang J, Sun J, Tian Z, Ji Y, Tang K, Zhou X, Cui G (2017). ACS Appl Mater Interfaces.

[30] Zhao H, Zhou X, Park S-J, Shi F, Fu Y, Ling M, Yuca N, Battaglia V, Liu G (2014). J Power Sources.

[31] Allmand A J, Cunliffe P W, Maddison R E W (1925). J Chem Soc, Trans.

