# Supplementary material for: High-speed C–H chlorination of ethylene carbonate using a new photoflow setup
Source: Beilstein J Org Chem. 2022 Jan 27;18:152–8. doi: 10.3762/bjoc.18.16 (PMC8805036; doi:10.3762/bjoc.18.16)
Supplement: File 1 — GC analysis and NMR spectra of the crude reaction mixture for the chlorination of compound 1. [file Beilstein_J_Org_Chem-18-152-s001.pdf]

BEILSTEIN JOURNAL OF ORGANIC CHEMISTRY

Supporting Information

for

High-speed C–H chlorination of ethylene carbonate using a new photoflow setup

Takayoshi Kasakado, Takahide Fukuyama, Tomohiro Nakagawa, Shinji Taguchi and Ilhyong Ryu

Beilstein J. Org. Chem. **2022,** *18,* 152–158. doi:10.3762/bjoc.18.16

GC analysis and NMR spectra of the crude reaction mixture for the chlorination of compound 1

License and Terms: This is a supporting information file under the terms of the Creative Commons Attribution License (https://creativecommons.org/licenses/by/4.0). Please note that the reuse, redistribution and reproduction in particular requires that the author(s) and source are credited and that individual graphics may be subject to special legal provisions.
The license is subject to the *Beilstein Journal of Organic Chemistry* terms and conditions: (https://www.beilstein-journals.org/bjoc/terms)

Table of contents

General information S2

Reference S2

GC Chart S2

Copies of NMR spectra S3

General information

GC analysis was performed on a Shimadzu GC-2014 instrument equipped with an FID detector using a J&W Scientific (Hongkong, China) DB-1 column under the following conditions: initial oven temperature was held at 40 °C for 5 min, the first ramp was 5 °C/min to 250 °C, which was held for 10 min. Yields were determined by using the percentage peak area method with compensation for the relative sensitivities of each component. ^{1}H NMR spectra were recorded with a JEOL JMN-ECS400 (400 MHz) and referenced to the solvent peak at 7.26 ppm. ^{13}C NMR spectra were recorded with a JEOL JMN-ECS400 (100 MHz) and referenced to the solvent peak at 77.0 ppm. Product **2** is a known compound [1] and the dichlorinated product **2'** is commercially available from Sigma–Aldrich Co. Inc. These compounds were identified by ^{1}H NMR and ^{13}C NMR analysis and comparison with the reported data.

Reference

1. Wang, W-.M.; Wang, W-.T.; Wang, M-.Y.; Gu, A-.L.; Hu, T-.D.; Zhang, Y-.X.; Wu, Z-.L. *Inorg. Chem*. **2021**, 60, 9122−9131

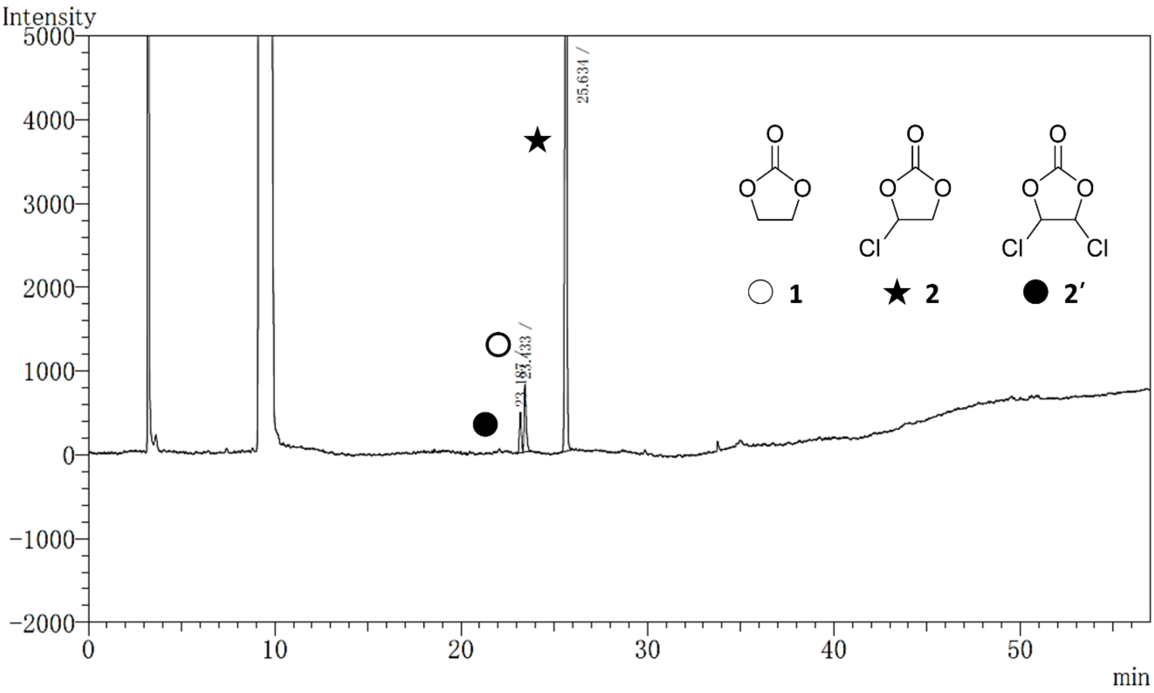

retention time (min)	area	height	concentration (area%)
23.187	3545	484	3.501
23.433	6022	805	5.948
25.634	91693	15698	90.552

Figure S1: GC analysis data of the crude product mixture from the C–H chlorination reaction of ethylene carbonate (**1**).

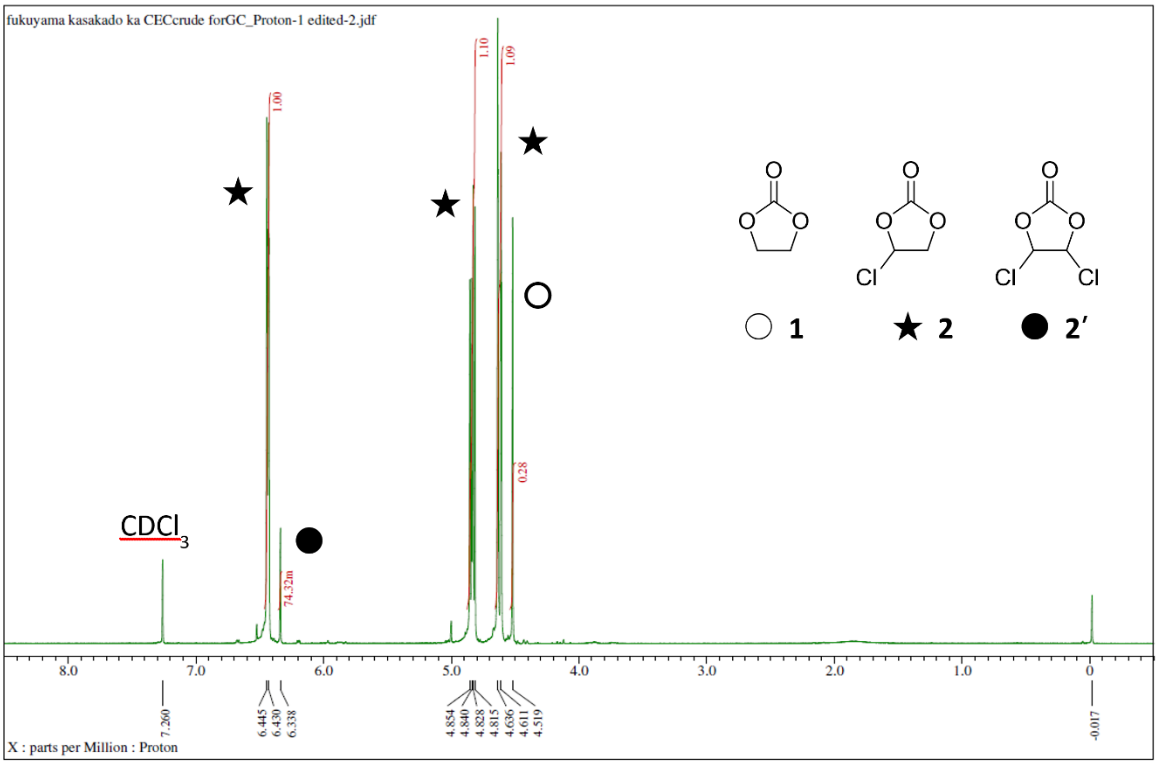

Figure S2: ^{1}H NMR spectrum of the crude reaction mixture from the C–H chlorination of ethylene carbonate (**1**) [1].

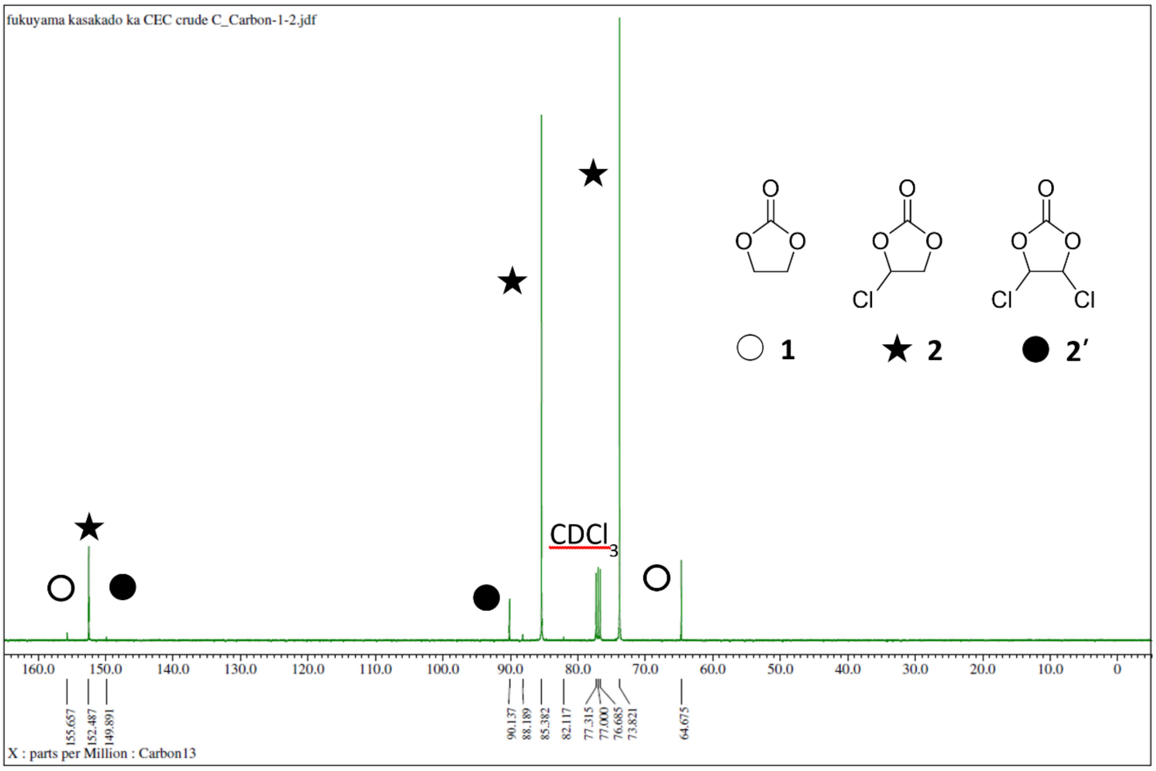

Figure S3: ^{13}C NMR spectrum of the crude reaction mixture from the C–H chlorination of ethylene carbonate (**1**) [1].
